# Effect of sowing proportion on above- and below-ground competition in maize–soybean intercrops

**DOI:** 10.1038/s41598-021-95242-w

**Published:** 2021-08-03

**Authors:** Yuanyuan Ren, Li Zhang, Minfei Yan, Yanjun Zhang, Yinglong Chen, Jairo A. Palta, Suiqi Zhang

**Affiliations:** 1 Geography and Environmental Engineering Department, Baoji University of Arts and Sciences, Baoji, 721013 China; 2 State Key Laboratory of Soil Erosion and Dryland Farming on the Loess Plateau, Institute of Soil and Water Conservation, Chinese Academy of Sciences and Ministry of Water Resources, Yangling, 712100 Shaanxi China; 3 State Key Laboratory of Soil Erosion and Dryland Farming on the Loess Plateau, Northwest A&F University, Yangling, 712100 Shaanxi China; 4 School of Plant Biology, The University of Western Australia, Perth, WA 6009 Australia; 5 CSIRO Agriculture and Food, Private Bag No. 5, Wembley, WA 6913 Australia

**Keywords:** Agroecology, Plant ecology

## Abstract

The relative contribution of above- and below-ground competition to crop yield under intercropping systems is critical to understanding the mechanisms of improved yield. Changes in the content of above- and below-ground biomass, leaf photosynthetic rate (Pn), leaf area index (LAI), chlorophyll meter reading (SPAD), diffuse non interceptance (DIFN), soil water storage (SWS), crop nitrogen (N), and phosphorus (P) uptake were examined in a 2-year trial of different maize–soybean intercropping systems on the Loess Plateau, China. Compared with the sole cropping system, shoot biomass of maize was increased by 54% in M2S2 and 62% in M2S4 strip intercropping treatment. The crop N and P uptake of maize increased significantly, by 54% and 50% in M2S2 and by 63% and 52% in M2S4 compared with their respective sole crop. LAI values of maize in intercropping systems were 14% and 15% for M2S2 and M2S4 less than that in the sole crop. The DIFN of intercropped maize was increased by 41% and 48% for M2S2 and M2S4 compared to monocrop. There were no significant differences in Pn and SWS in both crops between the two cropping systems. The contribution rate of DIFN in M2S2 and crop P uptake in M2S4 on the biological yield in intercropping system was the highest among all factors. We conclude that the sowing proportion affects above- and below-ground competition in maize–soybean intercropping systems.

## Introduction

The key to ensuring food security in more densely populated countries, such as China, is to improve crop yield on the existing cultivated land^1,2^. Modern agricultural production (sole crop) mode is characterized by a single form, and the pursuit of high input and high yield is known to lead to many ecological and environmental problems^3,4^. Intercropping systems, especially cereal–legume intercropping systems, have been proven to play a vital role in mitigating these problems. Intercropping cereal crops with legumes can increase the soil nitrogen availability through atmospheric nitrogen fixation, thereby reducing the dependence on nitrogen fertilizer and the risk of nitrogen losses through leaching^5^. The advantages of intercropping include increasing light, water, nutrients use efficiency, and reduction in competition from weeds and pressure from herbivores and pathogens^6^. Intercropping can also improve land-use efficiency^7^ and increase diversity in an agroecosystem, which is vital for stabilization of ecosystem productivity, especially when the climate is undergoing rapid changes^8^.

Interspecific interactions, including both above- and below-ground relationships, play prominent parts in determining the structure and dynamics of crop populations in agroecosystems^9,10^. Previous studies on the effect of above-ground interactions on the yield have been reported^7,11^. Interactions between species include interspecific facilitation and interspecific competition^12^. Competition for sunlight is perhaps one of the most important interspecific above-ground interactions in intercropping ecosystems^13^. The parameters related to sunlight use include leaf area index (LAI)^14^, chlorophyll meter reading (SPAD)^15^, diffuse non interceptance (DIFN) and leaf photosynthesis rate (Pn)^16^. In an intercropping system, taller crops seek more light and suffer slight competition when sharing light with companion crops^14^; also, for shorter crops, plant height increases and leaf photosynthesis rate decreases due to shading from taller crops^17^. The belowground competition for resources such as nutrients and water are also critical in improving crop yield under intercrops. Below-ground interspecies interactions contributed to the increased yields due to water movement in maize–pea intercropping systems^18^ and nutrient uptake in maize–soybean intercropping systems^16^ as a result of root growth^19,20^.

Such prior studies found that there were strong interactions between root and shoot competition^21^, below-ground competition usually affected the balance between the competing species more than above-ground competition^16,22^, and the contribution of above-ground interactions to crop growth was higher than that of below-ground interaction^13^. Previous research mainly evaluated the effects of above- and below-ground competition on the growth of intercropping system through separation treatments, such as no separation, above-ground separation, below-ground separation, above- and below-ground separation^23,24^. These studies typically consisted of separation experiments of shoots and roots under controlled environments^14,25,26^ or field conditions^13,16,27^ that possibly cause damage to the original structure of the soil layer, which can influence quantification of the relative contribution of the above- and below-ground competition to the yield of intercrops. In addition, previous studies only qualitatively reported the effects of above- and below-ground competition on the growth intercropping systems, without quantifying the influence of above- and below-ground competition to the growth of intercropping systems. Therefore, the contribution of the above- and below-ground competition to growth of intercropping systems in the field remains unclear. To resolve this question, we collected observations on the interaction between above- and below-ground of intercropped crops under different cropping systems in the field to quantify the sources of intercropping advantages. The objectives of this study were to (1) compare yield, above- and below-ground related parameters (Pn, LAI, SPAD, DIFN, soil water storage, nitrogen and phosphorus uptake) in different maize–soybean intercrops, and (2) quantify the relative contributions of above- and below-ground competition to intercrop system performance.

## Results

### Crop biomass, LER and WER

**Figure 1:**
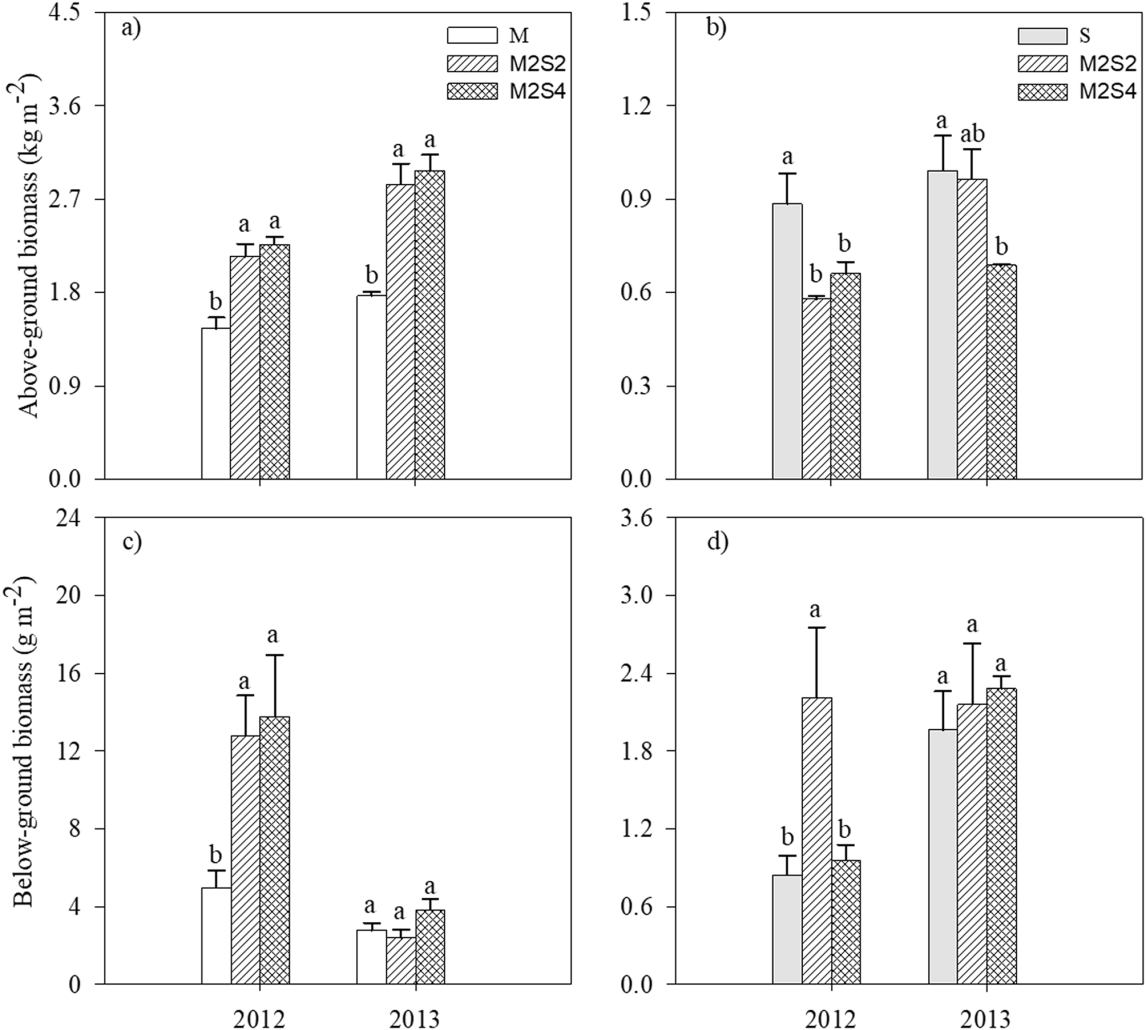
Above- and below-ground biomass of maize (**a**, **c**) and soybean (**b**, **d**) grown under sole cropping and intercropping systems in 2012 and 2013. Bars are means + standard errors. Bars with different letters are significantly different between cropping systems for each year (*P* < 0.05). M, sole-cropped maize; S, sole-cropped soybean; M2S2, two rows of maize intercropped with two rows of soybean; M2S4, two rows of maize intercropped with four rows of soybean.

**Figure 2:**
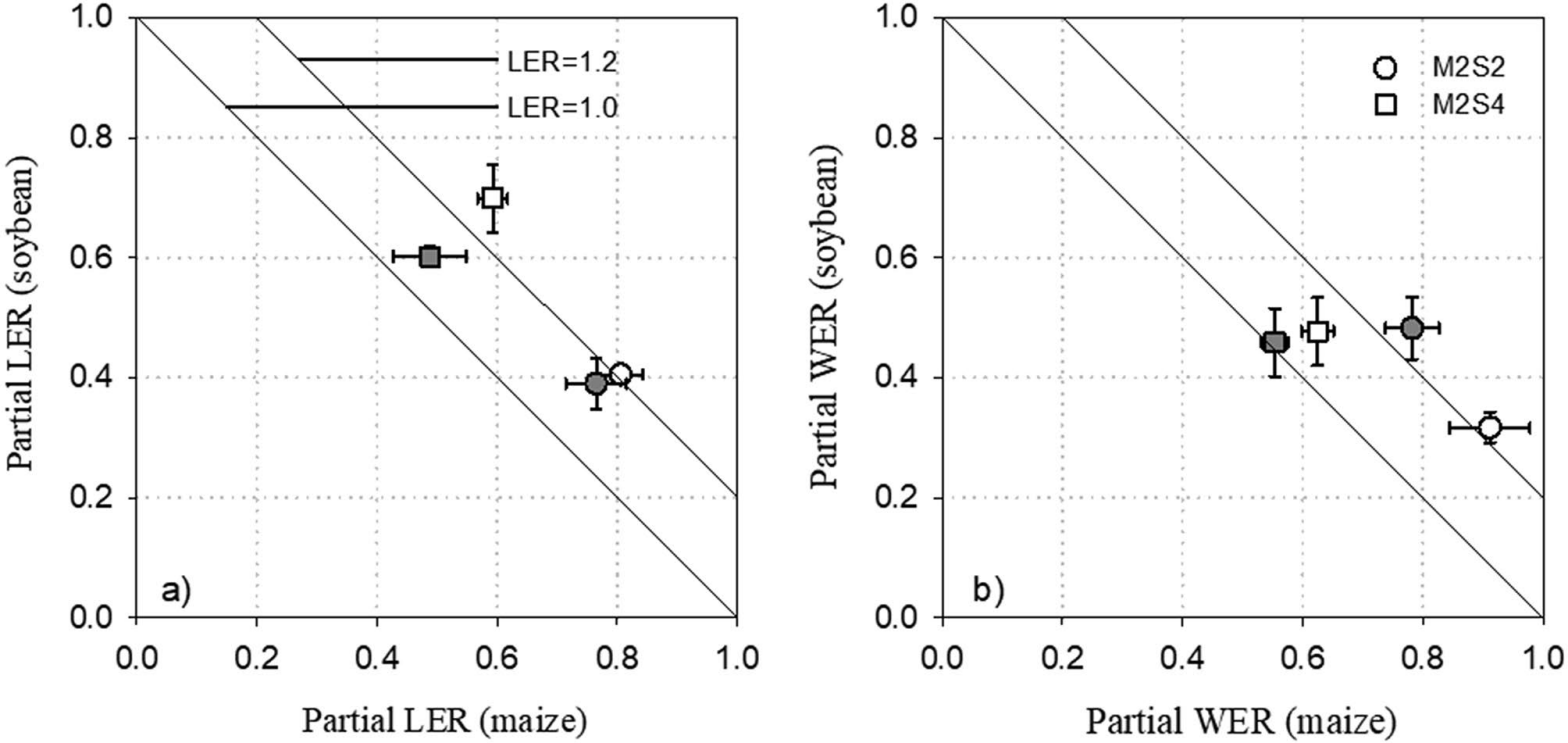
Partial land equivalent ratio (partial LER, **a**) and water equivalent ratio (partial WER, **b**) of the maize and soybean under intercrop systems in 2012 (white symbols) and 2013 (gray symbols) on the basis of yield. Bars are means ± standard errors.

Compared with the sole cropping system, the shoot biomass of the intercropped maize increased 54% and 62% for M2S2 and M2S4, respectively (Fig. 1a) whereas shoot biomass of intercropped soybean was no change or decrease (Fig. 1b). The root biomass of intercropped maize under M2S4 increased 38–178% compared with sole crop (Fig. 1c). The root biomass of soybean under M2S4 was no difference compared with sole-cropped soybean (Fig. 1d). Compared with the sole cropping systems, the land equivalent ratio (LER) based on the yield in intercropping systems increased 18% and 19% for M2S2 and M2S4, respectively (Fig. 2a), suggesting that maize–soybean intercropping systems have intercropping advantage in land-use efficiency. The water equivalent ratio (WER) in intercropping systems increased by 25% and 6% in M2S2 and M2S4, respectively, compared to monocrops (Fig. 2b), showing that intercropping can improve water use efficiency in maize–soybean intercrop.

### Above-ground interaction

**Figure 3:**
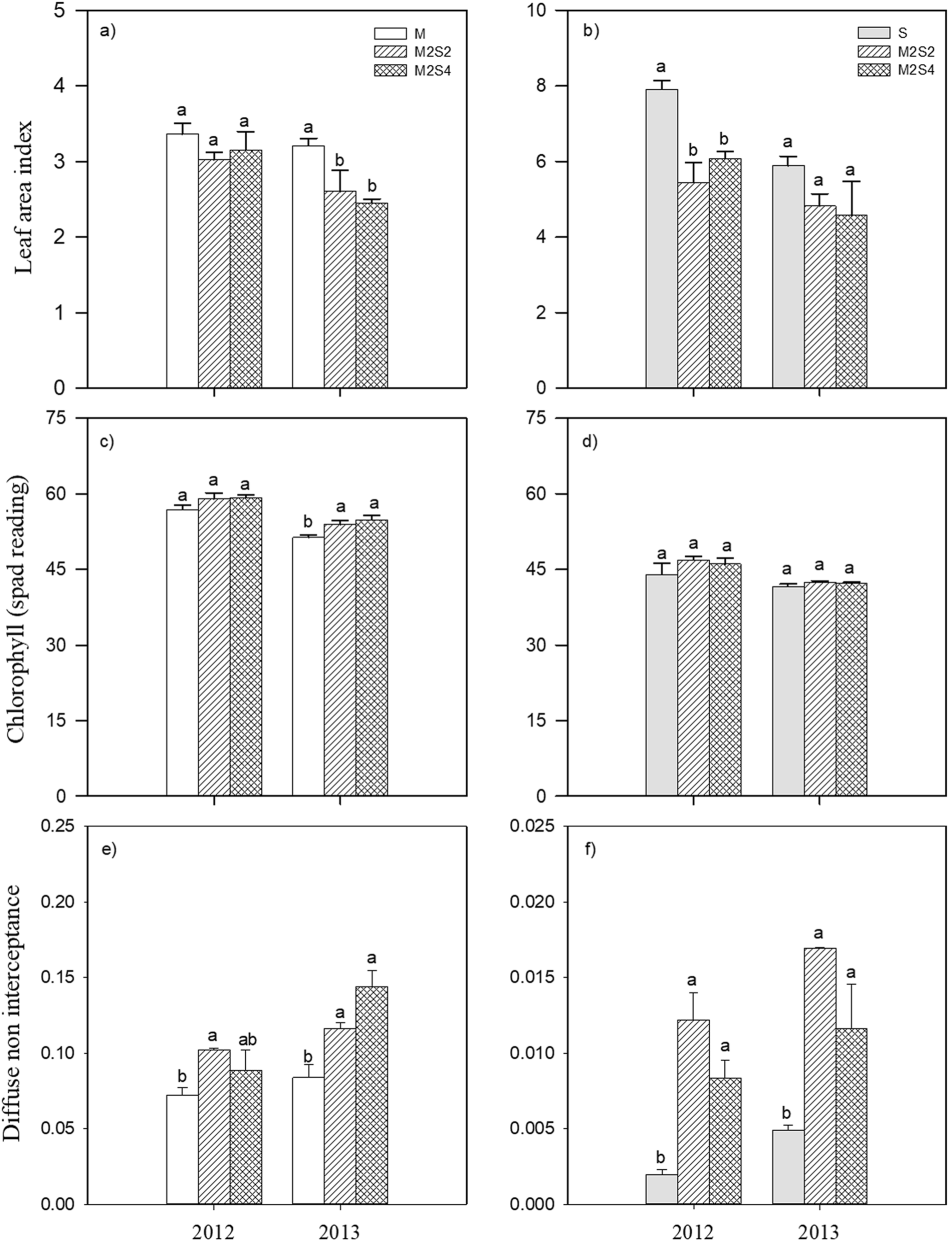
Leaf area index, chlorophyll (spad reading), diffuse non interceptance of maize (**a**, **c**, **e**) and soybean (**b**, **d**, **f**) grown under sole crop and intercrop systems in 2012 and 2013. Bars are means + standard errors. Bars with different letters are significantly different between cropping systems for each year (*P* < 0.05). M, sole-cropped maize; S, sole-cropped soybean; M2S2, two rows of maize intercropped with two rows of soybean; M2S4, two rows of maize intercropped with four rows of soybean.

There were no differences for the Pn of crops (maize and soybean) under sole cropping and intercropping systems in both years (Table 1). There were substantial variations among different cropping systems in the LAI values for maize and soybean in both years. The LAI of intercropped maize was reduced 14% and 15% for M2S2 and M2S4 compared to sole crop (Fig. 3a), and similarly, the LAI of intercropped soybean was reduced 25% and 23% for M2S2 and M2S4 compared to sole crop (Fig. 3b). The SPAD of intercropped maize was increased 4% and 5% for M2S2 and M2S4 compared to monocrop, and there was no difference for SPAD of soybean among different cropping systems (Fig. 3c, d). The DIFN of intercropped maize was increased 41% and 48% for M2S2 and M2S4 compared to monocrop, and DIFN of intercropped soybean was 4.82 and 3.30 times for M2S2 and M2S4 compared to monocrop (Fig. 3e, f).

**Table 1:** The leaf photosynthetic rate (Pn, μmol m^−2^ s^−1^) of maize and soybean under sole cropping and intercropping systems in 2012 and 2013.

Cropping system	Pn of maize		Pn of soybean	
	2012	2013	2012	2013
Sole crop	13.58a	18.50a	10.08a	7.21a
M2S2	17.44a	17.85a	9.06a	5.85a
M2S4	15.20a	21.05a	9.32a	6.04a
**ANOVA**				
Year	**		***	
Cropping system	ns		*	
Year × cropping system	ns		ns	

M2S2—two rows of maize intercropped with two rows of soybean; M2S4—two rows of maize intercropped with four rows of soybean. *, significant at 0.05 level; **, significant at 0.01 level; ***, significant at 0.001 level; ns, no significant difference. For each column, mean values indicated by different letters are significantly different at the 5% level using LSD.

### Below-ground interaction

**Figure 4:**
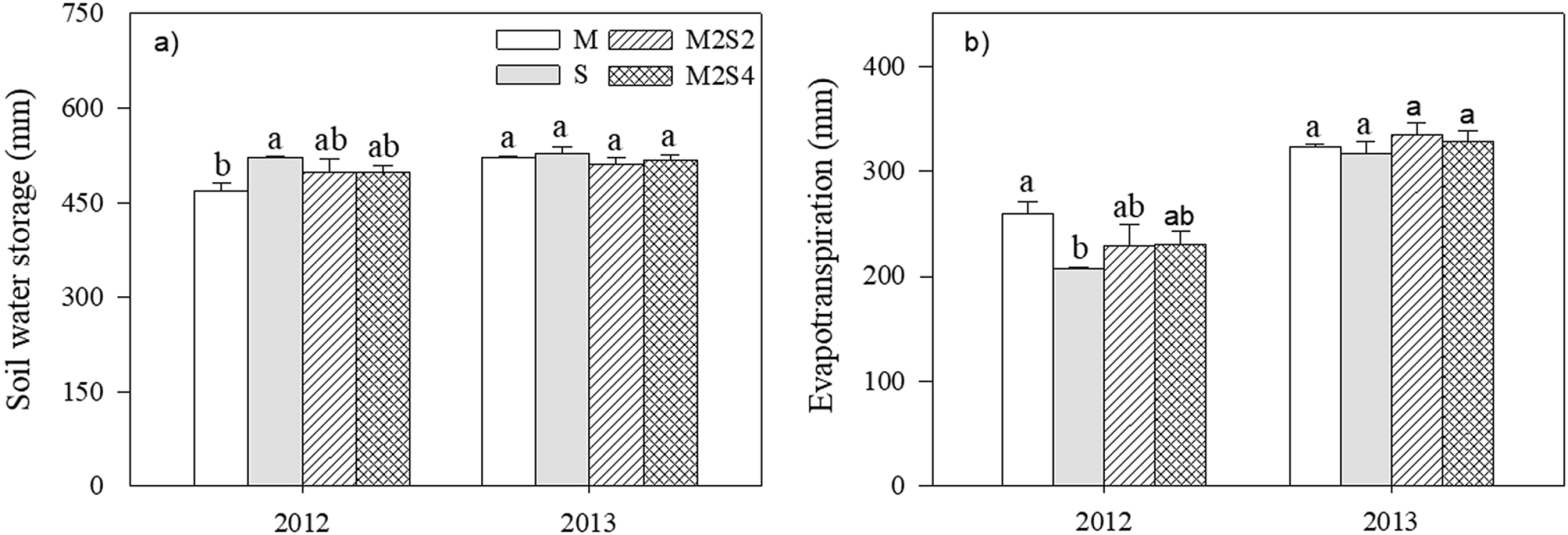
Soil water storage (**a**) and evapotranspiration (**b**) under sole cropping and intercropping systems. Bars are means + standard errors. Bars with different letters are significantly different between cropping systems for each year (*P* < 0.05). M, sole-cropped maize; S, sole-cropped soybean; M2S2, two rows of maize intercropped with two rows of soybean; M2S4, two rows of maize intercropped with four rows of soybean.

There was no difference in soil water storage (SWS) or evapotranspiration (ET) between intercropping and monocropping (Fig. 4). There was no difference for N concentration of maize, and for P concentration of maize between monocrop and intercrops except M2S4 in 2013 (Table 2).There was no difference in N and P concentration of soybean among different cropping systems (Table 2).The nitrogen (N) uptake of maize increased significantly (54% in M2S2 and 63% in M2S4) under intercrops compared to sole crop. The N uptake of soybean in intercrop decreased (22% in M2S2 and 35% in M2S4) compared to monocrop (Table 2). The variables of phosphorus (P) of maize coincided with the degree of nitrogen of maize. The P uptake of maize increased significantly (50% in M2S2 and 52% in M2S4) under intercrops compared to the sole crop. The P uptake of soybean in intercrop decreased 25.2% and 25.4% for M2S2 and M2S4 compared to monocrop. There were significant nutrient uptake advantages when maize intercropped with soybean compared to monocrop, showing that below-ground competition between intercropped crops plays an important role in the process of crop growth.

**Table 2:** N and P concentration and uptake of maize and soybean grown under sole cropping and intercropping systems in 2012 and 2013.

Cropping system	N concentration (%)		N uptake (kg ha^−1^)		P concentration (%)		P uptake (kg ha^−1^)	
	2012	2013	2012	2013	2012	2013	2012	2013
**Maize**								
Sole crop	1.38a	1.16a	200b	205b	0.11a	0.13a	15.8b	22.7b
M2S2	1.35a	1.18a	289a	337a	0.11a	0.12ab	23.2a	34.6a
M2S4	1.41a	1.15a	319a	343a	0.12a	0.10b	26.5a	31.0a
**Soybean**								
Sole crop	3.78a	3.17a	333a	294a	0.19a	0.21a	16.2a	19.7a
M2S2	3.41a	3.02a	197b	286a	0.16a	0.20a	9.01c	18.5a
M2S4	3.38a	2.70a	222b	186b	0.20a	0.19a	13.3b	13.2b
**ANOVA**								
Year	**		ns		ns		***	
Crop species	***		ns		***		***	
Cropping system	ns		ns		ns		ns	
Year × crop species	ns		ns		ns		ns	
Year × crop system	ns		ns		ns		ns	
Crop species × crop system	ns		***		ns		***	
Year × crop species × crop system	ns		ns		ns		ns	

M2S2—two rows of maize intercropped with two rows of soybean; M2S4—two rows of maize intercropped with four rows of soybean. *, significant at 0.05 level; **, significant at 0.01 level; ***, significant at 0.001 level; ns, no significant difference. The mean values indicated by a different letter in the same column are significantly different at the 5% level using LSD.

### Contribution to biological yield

**Figure 5:**
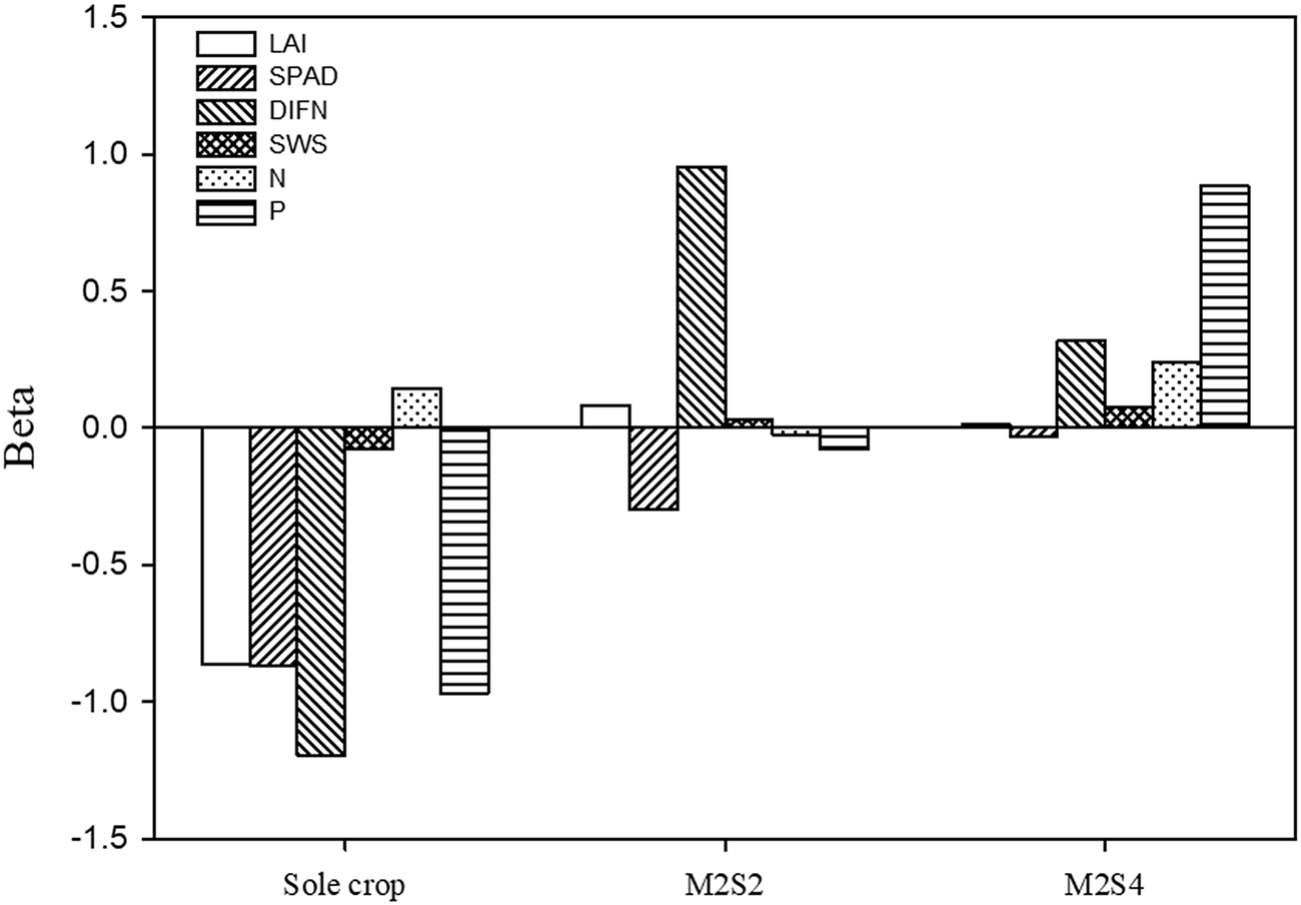
Contribution value of above- and below-ground interaction on the biological yield. M2S2, two rows of maize intercropped with two rows of soybean; M2S4, two rows of maize intercropped with four rows of soybean.

There was a correlation between LER and maize above-ground biomass (*R* = 0.589, *P* < 0.05) and soybean above-ground biomass (*R* = 0.672, *P* < 0.01), respectively (Table 3). The biomass of maize increased while that of soybean decreased in intercrops, which indicated that the advantage of intercropping in yield was mainly due to the increase of maize yield. In addition, there was no difference in ET between monocrop and intercrop, which improved the water use efficiency of intercropping system. In order to quantify the contribution of above- and below-ground interactions to biological yield, a relationship was obtained between biological yield and impact factors by linear regression. The biological yield (Y) is closely correlated with LAI, SPAD, DIFN, SWS, and the crop N and P uptake (Sole crop: Y =  − 1.67LAI − 0.50SPAD + 119DIFN − 0.01SWS + 0.008 N − 1.10P + 7.22 × 10^−7^X + 64, *R*^2^ = 0.96, *P* < 0.05; M2S2: Y = 0.62LAI − 0.44SPAD + 189DIFN + 0.01SWS − 0.004 N − 0.08P + 1.09 × 10^−7^X + 15.32, *R*^2^ = 0.99, *P* < 0.01; M2S4: Y = 0.08LAI − 0.05SPAD + 56DIFN + 0.04SWS − 0.03 N + 1.10P − 8.11 × 10^−8^X − 34.14, *R*^2^ = 0.99, *P* < 0.01). The yield in the sole cropping system was mainly determined by DIFN, as the contribution value of DIFN (|− 1.20|), and the second factor by crop P uptake (|− 0.97|) (Fig. 5), indicating that there were both above- and below-ground competitions. The yield in the intercropping system M2S2 was mainly determined by DIFN (|0.96|), mainly from the aboveground competition. The yield in the intercropping system M2S4 was mainly determined by the crop P uptake, as the contribution value of crop P uptake (|0.88|) was larger than that of other impact factors (Fig. 5), primary results from below-ground competition. Results clearly indicate that different planting patterns affect the above- and below-ground competition.

**Table 3:** The correlation between LER and above- and below-ground biomass.

	LER	Shootm	Shoots	Rootm	Roots
LER	1.00	0.59*	0.67**	0.03	− 0.24
Shootm	0.59*	1.00	0.34	− 0.29	0.05
Shoots	0.67**	0.34	1.00	− 0.33	− 0.10
Rootm	0.03	− 0.29	− 0.33	1.00	− 0.41
Roots	− 0.24	0.05	− 0.10	− 0.41	1.00

*, significant at 0.05 level; **, significant at 0.01 level. Shootm and Shoots represent the above-ground biomass of maize and soybean, respectively. Rootm and Roots represent the below-ground biomass of maize and soybean, respectively.

## Discussion

The maize–soybean intercropping enhanced land use efficiency 18% in M2S2 and 19% in M2S4, as indicated by LER (Fig. 2), showing that there was a greater biological efficiency in the maize–soybean intercropping system. There was a correlation between LER and above-ground biomass of maize and soybean, respectively (Table 3), indicating that yield was significantly positively correlated with the above-ground growth^28,29^. In addition, we found that maize yield had a significant positive correlation with the P uptake (*R* = 0.685, *P* < 0.05). The advantages of intercropping therefore appear to be related primarily to cereal, which is the dominant species with higher competitiveness compared with legumes in the cereal–legume intercrop^30^.

### Above-and below-ground interaction

The advantage of intercropping is due to above- and below-ground interactions. This study showed no difference in the photosynthetic rate of both soybean and maize between intercropping and sole cropping systems. Similar results were found for soybean intercropped with maize^4^ or wheat^31^. LAI has been shown to be an effective method by which to evaluate light absorption, and the photosynthetic rate of a crop is directly affected by the LAI^29,32^. We found that the LAI of intercropped soybean and maize are lower than that of the corresponding sole crop, indicating that the two species restrained each other in LAI of the competing crop when intercropped^33^. The DIFN was significantly improved under intercrops, showing that intercrop increased light transmission of the canopy and decreased the waste of light energy^34^. The maize–soybean intercrop improved the N and P uptake in maize, and there was no change for N and P concentration in maize, showing that N and P uptake improved were the consequence of increased in biomass production. This is consistent with the results of previous research on maize intercropping with faba bean^20^, soybean^16^, and cowpea^35^. Nitrogen leached from the root zone of soybean could be absorbed by nearby roots of maize, indicating that intercropping improve nitrogen fertilization absorption^36^. The maize–soybean intercrops promoted nitrogen fixation of soybean from air, and nitrogen could transfer from soybean to maize in intercrops, which would be beneficial to improve nitrogen fertilization absorption of maize. The soybean could have the ability to acidify the rhizosphere by releasing protons and mobilizing insoluble soil phosphorus by exuding malate and citrate, which could increase phosphorus fertilization absorption of maize.

### Contribution to biological yield

Some studies implied that below-ground competition have contributed greatly to intercrop advantages than above-ground interaction by shoot or root separation experiment in maize–soybean intercrops^16^. Some studies showed that intercrop advantages result from above-ground competition than below-ground competition by row spacing and root separation experiments in maize–soybean intercrops^13^. All of the previous studies on the impact of above- or below-ground interactions to intercropping advantage were studied by qualitative description. In our study, we quantified the effects of above- (Pn, SPAD, DIFN, and LAI) and below-ground (SWS, crop N and P uptake) interactions on intercropping systems by calculating the contribution rate.

The narrow-wide row configuration affect microclimate environment of interspecies, crop growth and yields because of different space and time for crop production^37^. We found that the above- and below-ground factors contribute to yield advantage in the order of DIFN > SPAD > LAI > P > SWS > N in 2:2 planting pattern, and P > DIFN > N > SWS > SPAD > LAI in 2:4 planting pattern. This shown that different intercrop pattern has an impact on crop yields on account of the above- and below-ground competition among intercropping systems^13^. For above-ground interaction, the row planting patterns affect the light transmission rate of intercropping species^38^, because close planting between different crops always causes mutual shading, especially for the shading of tall crops over short crops in intercropping systems, and then inevitably affect crop radiation interception^39^, light interception^40^, and photosynthetically active radiation transmittances^13^. This result is consistent with the previous studies that intercropping patterns affects light use efficiency and light interception by regulating intraspecific and interspecific competition and compensating interactions^40^. We found the most important factor contributes to yield under M2S2 is above-ground competition for light, as contribution value of DIFN. Previous studies also found that above-ground interactions have more contributions to intercrop advantage than below-ground interactions in different intercropping systems^13^. Compared to M2S4 intercrop, above-ground interactions under M2S2 due to plant canopy structure, such as mutual shading, greater affect light interception and light transmission rate.

For below-ground interaction, the sowing proportions has a significant effect on the crop N and P uptake, because of the different root growth and distribution of crops in maize–soybean^41^, proso millet-mung bean^42^ intercropping system. The greater lateral root growth, as well as compatibility of spatial root distribution of the component species in intercropping system has a positive effect on crop growth and yield^43^. The N and P uptake of wheat in wheat–maize intercropping system improved by approximately 50%^44^. This sowing proportions in intercrop increases crop root growth and soil volume to capture more water and nutrient in the soil profile^41^. The most important factor contributes to yield under M2S4 intercrop is below-ground competition for P nutrient, as contribution value of crop P uptake, which is consistent with previous studies that yield in intercrop may be more influenced by below- than above-ground crop interactions^27,45^. Intercropping advantage mainly comes from belowground interaction, including maximized soil nutrients utilization, due to the mingling of the roots of both crops^23^, water movement and root overlapping activity^27^, greater below-ground space, and a longer life span of crop roots^20^. We found that increased crop P uptake was the main factor leading to the advantages of intercropping, as the contribution rate of crop P uptake on the biological yield was the highest among below-ground factors, which is consistent with the results observed for cowpea–maize^35^, barley–legume^46^, chickpea–wheat^47^, maize–alfalfa^48^, and common bean–maize^49^ intercropping systems. Increased P uptake in intercrops was mainly due to increased root length density^41^, increased P availability by rhizosphere pH change^35,48^ or root-induced alkalization^49^, and facilitated organic P utilization^50^ because the rhizosphere is acidified through the roots^51^.

## Conclusions

Land equivalent ratio were 1.18 and 1.19 for 2:2 and 2:4 intercrops, showing that there were intercropping advantage in land-use efficiency for maize intercropped soybean. The interactions between above- and/or below-ground components was surveyed to improve our understanding of the performance of intercropping. The sowing proportions affects above- and below-ground competition in maize–soybean intercropping systems. The yield under 2:2 and 2:4 intercropping system was mainly determined by diffuse non interceptance, and crop phosphorus uptake, respectively. Thus, above-ground competition was a more important contributor to intercrop advantages than below-ground competition under 2:2 intercrop system, and below-ground competition was significantly higher than above-ground competition to intercrop advantages under 2:4 intercrop system.

## Methods

### Site description

Field experiments were conducted at the Changwu Experimental Station (35° 12′ N, 107° 40′ E, altitude 1200 m) located in Shaanxi Province, China. The experimental site was in the typical dryland farming area on the Loess Plateau. Annual precipitation in the area averaged 582 mm between 1957 and 2013, with a mean annual temperature of 9.7 °C over that period. Rainfall and temperature during the two study years are shown in Fig. S1. Soils were generally of the Calcaric Regosol group, according to the FAO/UNESCO soil classification system^52^, and were composed of 4% sand, 59% silt, and 37% clay^53^. The 0–20 cm soil properties were the following: pH, 8.4; organic matter content, 11.8 g kg^−1^; total N content, 0.87 g kg^−1^; and Olsen-P, 14.4 mg kg^−1^.

### Experimental design and field management

Two-year experiment was arranged in a randomized complete block design with three replicate plots during 2012 and 2013 growing seasons^25,54^_ENREF_53. The study was conducted using the soybean cultivar (*Glycine max* L.) cv. Zhonghuang 24 and the maize cultivar (*Zea mays* L.) cv. Zhengdan 958 grown in cereal–legume agricultural systems. Zhonghuang 24 was bred from Jilin 21 and fendou 31 × Zhongdou 19 (deposition number 2008003); Zhengdan 958 was the offspring of inbred Zheng 58 and Chang 7-2 (deposition number 20000009), which are approved in China. The cropping system treatments were as follows:

1. Sole-cropped soybean (S).
2. Sole-cropped maize (M).
3. Two rows of maize intercropped with two rows of soybean (M2S2).
4. Two rows of maize intercropped with four rows of soybean (M2S4).

Each plot measured 6 m × 4 m, with row spacing of 50 cm for maize and soybean both in sole crops and intercrops. Individual plants were spaced at 22 cm and 19 cm for maize and soybean, respectively, with one plant per stand for maize and two plants per stand for soybean to attain densities of 90,000 and 210,000 plants ha^−1^, respectively. In 2012, seeds of maize and soybean were sown on 25 April and harvested on 28 September, and in 2013, seeds were sown on 20 April and harvested on 25 September. Before sowing, basal fertilizer was applied at a rate of 90 kg N ha^−1^ as urea (46% N) and 150 kg P_2_O_5_ ha^−1^ as superphosphate (12%, P_2_O_5_), and then additional fertilizers were uniformly spread in each plot, which were then ploughed into the 0–30 cm soil layer using a rotary tiller. All of the plots received 67.5 kg N ha^−1^ as urea at the bell and silking stages using a hole-seeding machine. No irrigation was applied, and weeds were removed by hand when sighted. The research on plants complied with relevant institutional, national, and international guidelines and legislation.

### Above- and below-ground measurements

The Pn was measured with a LI-6400 portable photosynthesis system (LI-COR Inc., Lincoln, NE, USA) from 9:00 to 11:00 h at 120 days after sowing, which corresponds to the milk stage in maize and full seed stage in soybean^7,13^. We measured photosynthesis of ear leaves of maize, the first spreading leaves at the top of soybean in both the sole crops and intercrops. The Pn values were calculated as the sum of the mean readings for five leaves in each plot. The LAI values, DIFN were recorded using a Plant Canopy Analyzer (Li-2200, LiCor Inc., Lincoln, NE, USA) without direct sunlight at milk stage of maize. One above-canopy measurement and three below-canopy measurements at the soil surface were taken for four replicates in each plot. SPAD were collected using a hand-held dual wavelength meter (SPAD 502, Chlorophyll meter, Minolta Camera Co., Ltd., Japan) at milk stage of maize. Measurements were taken midway along the ear leaves of maize and the first spreading leaves at the top of soybean from five adjacent plants at the center of row in each plot.

The SWS was measured gravimetrically using a soil auger at 10 cm intervals over a depth of 100 cm and at 20 cm intervals over a depth of 200 cm at milk stage of maize for three replicates in each plot. The SWS was calculated for each plot in the 0–200 cm soil profile for the soil moisture using the following formula: SWS = SWC × SD × SBD, where SWC represents soil water content, SD represents soil depth, and SBD represents soil bulk density. Apparent water use during crop growth season was expressed as evapotranspiration (ET), which was determined according to the following formula: ET = ΔSWS + P, where ΔSWS is the change in soil water storage in the top 200 cm and P is the rainfall (mm) between planting and at milk stage in maize. The six adjacent plant samples were collected at milk stage of maize in the middle two rows of each plots (Fig. S2). The sampling included shoots and roots of maize and soybean. At the cotyledonary node, above-ground parts were separated from below-ground parts. Soil core samples (9 cm diameter × 15 cm) at the intra-row of crop were collected to a depth of 100 cm using an auger and separated in 10-cm sections to determine the root growth in sole-cropping and intercropping systems. The samples were exposed to 105 °C for 30 min and then dried to a constant weight at 75 °C. The oven-dried samples were put in small plastic bags after grinding. The study of N and P uptake are the most common among mineral elements^55,56^. Concentrations of N and P in the plant dry matter were determined after digestion with H_2_SO_4_ and H_2_O_2_; N concentration was measured according to the Kjeldahl method^20^, whereas P concentration was measured by the molybdenum-antimony anti-spectrophotometric method^16^. Crop N and P uptake were calculated by the actual above-ground biomass multiplied by plant tissue N and P concentrations. Grain yield was estimated at harvest from 6 m^2^ for maize and soybean based on the average of three plot replicates.

### Data analysis

The LER for assessment of land use advantage. LER is sum of ratio of intercrop to sole crop for maize and soybean yield^57^:

$$ LER = LER_{m} + LER_{s} ,\;LER_{m} = \frac{{Y_{im} }}{{Y_{sm} }}, \;LER_{s} = \frac{{Y_{is} }}{{Y_{ss} }} $$

where *LER*_*m*_ and *LER*_*s*_ are patial *LER* for maize and soybean, respectively. *Y*_*im*_ and *Y*_*is*_ are yields of maize and soybean under intercrops, respectively. *Y*_*sm*_ and *Y*_*ss*_ are the yield of maize and soybean under sole crop, respectively.

The water equivalent ratio (WER) was calculated to measure water use advantage of intercropping^58^:

$$ WER = WER_{m} + WER_{s} ,\;WER_{m} = \frac{{Y_{im} /ET_{im} }}{{Y_{sm} /ET_{sm} }},\;WER_{s} = \frac{{Y_{is} /ET_{is} }}{{Y_{ss} /ET_{ss} }} $$

where *WER*_*m*_ and *WER*_*s*_ are patial *WER* for maize and soybean, respectively. *ET*_*im*_ and *ET*_*is*_ are *ET* of maize and soybean under intercrops, respectively. *ET*_*sm*_ and *ET*_*ss*_ are the *ET* of maize and soybean under sole crop, respectively.

All analyses were conducted in SPSS Statistics 17.0 (SPSS Inc., Chicago, IL, USA). Treatment means showing significant differences among different cropping systems were separated using one-way ANOVA or least significant difference (LSD) at a threshold of 5% to compare the effect of yield, above- and below-ground related parameters (Pn, LAI, SPAD, DIFN, SWS, N and P uptake) in different maize–soybean intercropping. The variation in Pn, LAI, SPAD, DIFN, SWS, N, and P uptake of crop, and the effects of cropping system × year were made using Univariate General Linear Models. Pearson’s correlation test was used to analyze between LER and above-and below-ground biomass of maize and soybean. The effects of above- and below-ground factors on biological yield were quantified, by calculating the contribution value of some key factors to yield. The effects of between above- (LAI, SPAD, DIFN) and below-ground (SWS, N and P uptake) competition on the biological yield and contribution rate were conducted by the linear regression model^59^:

1 $$ Y = \beta_{0} LAI + \beta_{1} SPAD + \beta_{2} DIFN + \beta_{3} SWS + \beta_{4} {\text{N}} + \beta_{5} {\text{P}} + \beta_{6} X + \beta_{7} $$

where *Y* represents biological yield, *LAI* represents leaf area index, *SPAD* represents chlorophyll, *DIFN* represents diffuse non interceptance, *SWS* represents soil water storage, N represents crop nitrogen uptake, P represents crop phosphorus uptake, *X* represents interaction for *LAI*, *SPAD*, *DIFN*, *SWS*, N, and P, and *β*_0_, *β*_1_, *β*_2_, *β*_3_, *β*_4_, *β*_5_, *β*_6_ and *β*_7_ represent the fitted parameters. The standard regression coefficients (Beta) of *LAI*, *SPAD*, *DIFN*, *SWS*, N, and P were determined on the basis of Eq. (1) to split their influence on the biological yield by the following equations:

2 $$ \beta_{0}^{\prime } = \beta_{0} \times \left( {LAI^{\prime } /Y^{\prime } } \right) $$

3 $$ \beta_{1}^{\prime } = \beta_{1} \times \left( {SPAD^{\prime } /Y^{\prime } } \right) $$

4 $$ \beta_{2}^{\prime } = \beta_{2} \times \left( {DIFN^{\prime } /Y^{\prime } } \right) $$

5 $$ \beta_{3}^{\prime } = \beta_{3} \times \left( {SWS^{\prime } /Y^{\prime } } \right) $$

6 $$ \beta_{4}^{\prime } = \beta_{4} \times \left( {{\text{N}}^{\prime } /Y^{\prime } } \right) $$

7 $$ \beta_{5}^{\prime } = \beta_{5} \times \left( {{\text{P}}^{\prime } /Y^{\prime } } \right) $$

where *β*_0_′, *β*_1_′, *β*_2_′, *β*_3_′, *β*_4_′, and *β*_5_′ represent the standard regression coefficients for *LAI*, *SPAD*, *DIFN, SWS*, N, and P. *LAI*′, *SPAD*′*, DIFN*′*, SWS*′, N′, and P′ represent the standard deviations for *LAI*, *SPAD*, *DIFN*, *SWS*, N, and P. *Y*′ is the standard deviation for the modeled biological yield.

## Supplementary Information

Supplementary Figures.
