# Supplementary material for: Effect of sowing proportion on above- and below-ground competition in maize–soybean intercrops
Source: Sci Rep. 2021 Aug 3;11:15760. doi: 10.1038/s41598-021-95242-w (PMC8333320; doi:10.1038/s41598-021-95242-w)
Supplement: Supplementary file 1 — Supplementary Figures. [file 41598_2021_95242_MOESM1_ESM.docx]

Supplementary Information

**Effect of sowing proportion on above- and below-ground competition in maize–soybean intercrops**

Yuanyuan Ren, Li Zhang, Minfei Yan, Yanjun Zhang, Yinglong Chen, Jairo A Palta, Suiqi Zhang*

* [sqzhang@ms.iswc.ac.cn](mailto:sqzhang@ms.iswc.ac.cn)

**Figure S1.**


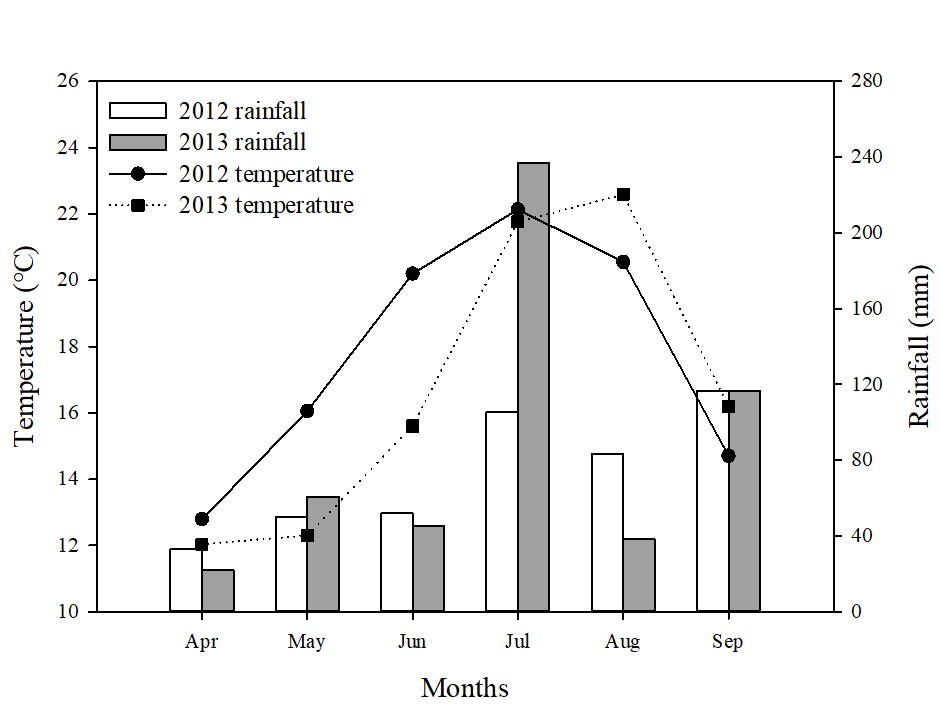


**Figure S1.** Monthly rainfall and mean temperature at the Changwu Experimental Station, China, in 2012 and 2013.

**Figure S2.**


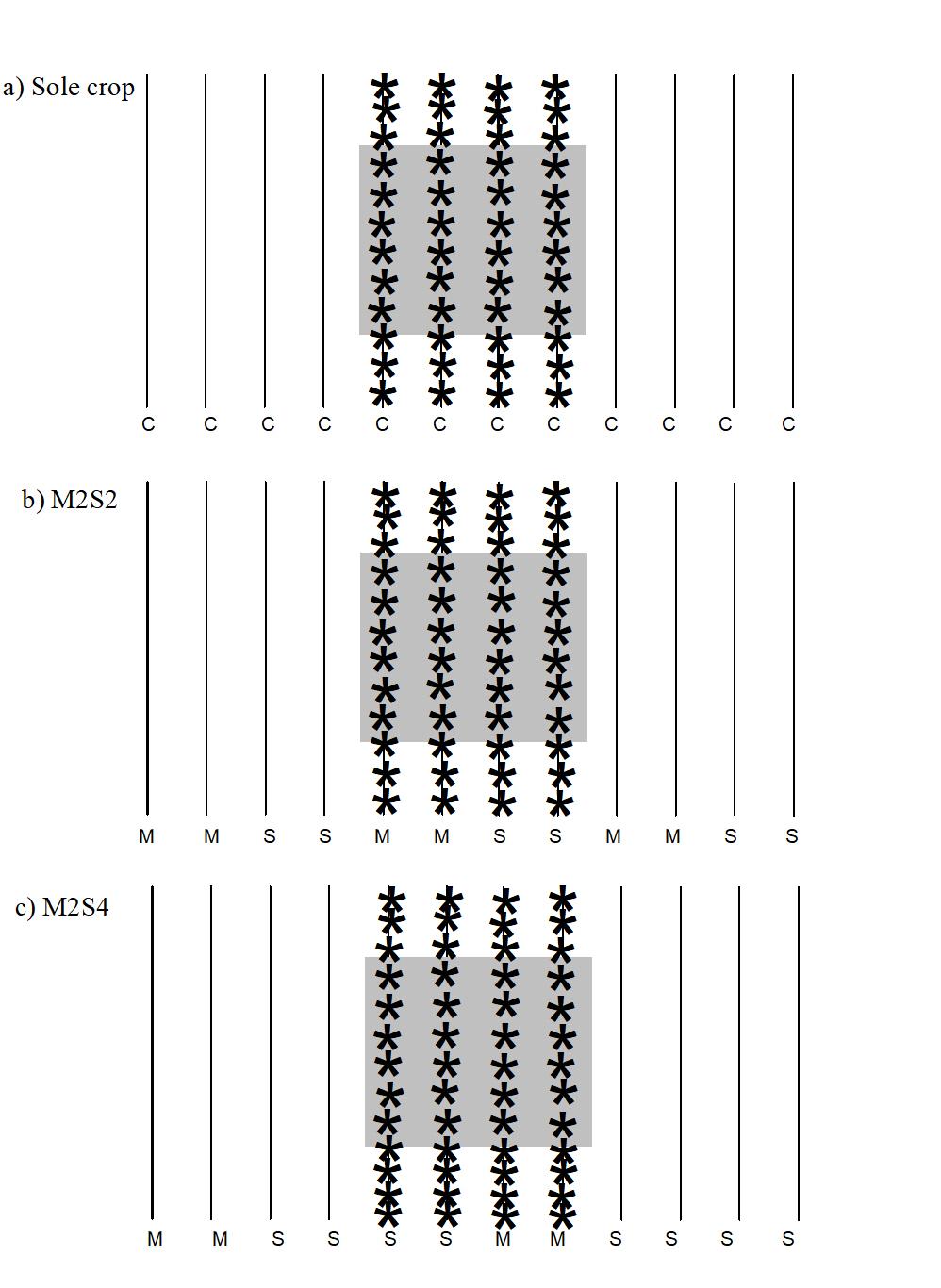


**Figure S2.** Biomass samples position

M2S2, two rows of maize intercropped with two rows of soybean; M2S4, two rows of maize intercropped with four rows of soybean; C, sole- cropped maize or soybean; M, maize; S, soybean.
